# *In Vitro* Inhibitory Effect of Clove Essential Oil and Its Two Active Principles on Tooth Decalcification by Apple Juice

**DOI:** 10.1155/2012/759618

**Published:** 2012-08-21

**Authors:** Charu M. Marya, Gunjan Satija, Avinash J., Ruchi Nagpal, Rohtash Kapoor, Aijaz Ahmad

**Affiliations:** ^1^Department of Public Health Dentistry, Sudha Rustagi College of Dental Sciences and Research, Faridabad, Haryana, India; ^2^Department of Prosthodontics, Maharaja Ganga Singh Dental and Research College, Sri Ganga Nagar, Rajasthan, India; ^3^Department of Biosciences, Jamia Millia Islamia, New Delhi, India

## Abstract

The dental erosion or decalcification of enamel is a significant clinical problem. Apple acidic beverages are thought to increase the potential for dental erosion. The purpose of this *in vitro* study was to investigate the effect of clove essential oil (CEO) and its active principles on tooth decalcification of apple juices. On GC-MS analysis, CEO showed a high content of eugenol (58.29%) and eugenyl acetate (19.10%). Teeth specimens were randomly divided into 5 treatment groups: control, CEO, eugenol, eugenyl-acetate, and fluoride. The specimens were exposed for 24 h and were analyzed for calcium contents using Inductively Coupled Plasma with Optical Emission Spectroscopy (ICP-OES). Data were analyzed using student *t*-test (*P* < 0.05). CEO, eugenol, and eugenyl-acetate significantly decreased the decalcification of tooth by the apple juice to only 17, 24, and 21 mgL^−1^, respectively. Hemolytic activity on human erythrocytes was studied to exclude the possibility of further associated cytotoxicity. It was observed that the CEO and its two lead molecules inhibit the decalcification and/or promote the remineralization caused by the apple juices. The effect of the test compounds appears to be distinct like that of fluoride treatment. CEO may, therefore, serve to be a promising adjunct to fluoride in the treatment of root caries during minimally invasive therapy.

## 1. Introduction 

As the mankind is evolving there has been drastic changes occurring in the dietary pattern as well. The diet we are consuming has become more refined with increased access to readymade fruit juices and high frequency of snacking. Also there has been substantial increase in consumption of carbonated beverages and fruit drinks [1]. Since recently there has been considerable emphasis on “healthy food and healthy eating.” Fruit juices have been widely marketed and promoted as “healthy drinks.” However, claims of safety of fruit juices for teeth are unsubstantiated due to inadequate report in the literature [2]. Apple juices are consumed globally but universally assumed to be innocent as to their effects on the mouth. Apple juices contain acids and sugars that can dissolve the hard structures of the teeth (the enamel and any exposed roots), leaving the inner parts of teeth exposed, which leads to sensitivity. Fruit juices are examples of foods that contain a mixture of reducing and nonreducing sugars (fructose, sucrose, and glucose), with the concentration varying according to the type and maturation status of the fruit. Such sugariness, coupled with an acidic nature, has caused fruit juice to be cited as a risk factor for dental decay [3]. Walsh stated that the dark cola drinks are the worst offenders when it comes to dental erosion; some highly acidic juices can do more damage to your teeth than other soft drinks [4]. Within the last few decades, decalcification of the teeth has become the major problem among all the age groups. The erosive effect of fruit juices have been recognized for a long time as evident in the various studies [5] who reported tooth decalcification due to excessive fruit juice consumption. Acidic foods and beverages can affect natural teeth, and chronic exposure often leads to the development of dental erosion, abrasion, and decay. There has been increased interest in determining some physical and chemical properties of fruit juices, such as endogenous pH, acidity, and total soluble solid content (TSSC), as well as their effects on dental erosions or decalcification of teeth. The acidity of a composition may be expressed in terms of titratable acidity, which is a measure of the percent weight of acid present in a solution. One of the important strategies regarding preventive therapies for dental erosions and decays is to prevent the dental erosion or to promote remineralization of demineralized teeth [6]. Therefore, there exists a clear demand of more effective treatment of tooth erosion. Natural products have been used as folk-medicines for thousands of years and are promising sources for novel therapeutic agents [7]. Majority of the studies of natural products in the field of oral health have focused on their antimicrobial activities [8, 9]. Very few reported on the effects of natural products or phytochemicals on the inhibition of dental erosion or decalcification processes of dental hard tissues [10].

Clove (*Syzygium aromaticum*) is time-honored for its medicinal properties; however, its medicinal characteristics are used only in “Ayurvedic medicines.” The principal phenolic components of clove essential oil, eugenol, and eugenyl acetate have been shown to change some physical properties of resin composite such as the adverse effect on surface roughness [11–13], transverse strength [14], and surface hardness [12, 15]. However, clove oil is supposed to cause serious problems like sore throat, vomiting, cytotoxicity, kidney failures, and/or damage to the liver, seizures, difficult breathing, and others if used in higher doses. Therefore, in the present study small doses showing least cytotoxic effects of this oil have been used.

The current study tests the hypothesis that clove essential oil and its lead molecules may positively inhibit the decalcification of teeth caused by the most widely used apple beverage, which offers a potential novel natural therapy for dental problems. In addition to this, clove essential oil was analysed by GC-MS analysis and the hemolytic activity of the test oil, and its lead molecules was included to exclude the possible cytotoxicity of these compounds.

## 2. Materials and Methods

The commercially available clove seeds were subjected to hydrodistillation in a Clevenger apparatus for 3 h. The essential oil (volatile fraction) was separated and stored at 4°C. Eugenol and eugenyl acetate were purchased from Sigmae Aldrich (USA). All inorganic chemicals were of analytical grade and procured from E. Merck (India). 

A pilot survey was conducted to know which fruit juice is most commonly consumed among the general population, which showed that among commercially available fruit juices the most commonly consumed were Apple, Guava, and Litchi juices. Among these three, apple juice is more acidic than guava juice (pH = 5.5) and litchi juice (pH = 5). Therefore, apple juice was considered in this study. 

### 2.1. Physiochemical Properties

Determination of endogenous pH, TA, and sugar levels was undertaken in samples of commercially available apple fruit juice. Each analysis was made in triplicate. Data were collected by a single calibrated examiner, recorded on study-specific charts.

The intrinsic pH of the apple juice was measured prior to the consumption. The pH of the apple juice was determined using a pH meter (Consort C832 Multiparameter analyzer) placed directly into the solution. Fifty milliliters of the juice was placed in a beaker, the pH meter electrode was immersed in the juice, and the reading was recorded. The recordings were taken at the room temperature. 

Titratable acidity is the amount of 0.1 N KOH solution needed for the product to reach a neutral pH or a pH value above it. Titratable acidity of the apple juice was measured according to the method adopted by the Association of Official Analytical Chemists [16], A 10 mL aliquot of the diluted product was titrated (10% solution of the sample) with the 0.1 N KOH solution until the substance reached a pH value of 7, corresponding to the endpoint of the phenolphthalein. Readings were done with a pH meter (Consort C832 Multiparameter analyzer). When this value was reached, the spent KOH volume was recorded and the acidic percentage of the substance was calculated using the following equation, with the result being expressed as percentage of citric acid,

(1) Acidity  (%citric  acid) =V×Nap×F×  meq-g  (citric  acid)×100/Sample,

where *V* = KOH volume; Nap = Normal concentration of the KOH base; *F* = Normality correction factor; meq-g = miliequivalent per gram of citric acid; Sample = volume of the medicine. The process was repeated on 3 different containers of the apple beverage. Also, the juice was assayed in triplicate after 60 minutes of vigorous stirring to remove any carbonation.

Reducing sugars (e.g., glucose), nonreducing sugars (e.g., sucrose), and total sugars were measured according to the method adopted by the Association of Official Analytical Chemists [16], and the results were expressed in g/mL.

**Reducing Sugars**

For determination of the reducing sugars, 5 mL of the apple juice was diluted in 50 mL of distilled water. This solution was heated in water bath for 5 min and, after cooling and filtering, its volume was completed to 100 mL with distilled water. Next, 10 mL of the Fehling's solution was mixed with 3 drops of 1% blue methylene. This mixture was titrated under warming blanket with the previously prepared sample solutions until the reducing sugars present in the sample reduced completely the Fehling solution, as demonstrated by color change from blue to colorless, and by the formation of a brick red precipitated. The measuring unit for reducing sugars is expressed as gram of glucose per 100 mL of the sample and the percentage of reducing sugars was calculated by the following equation:

(2) %  of  reducing  sugars  in  glucose  =  100  ×  V  ×  CfSv  ×  Tv,

*V* = Volume (mL) of the sample solution, Cf = Calibration factor of the Fehling solution, *S*_*v*_ = Volume of the sample used for preparation of the solution, and *T*_*v*_ = Volume of the sample solution used in the titration.

**Total Sugars**

In this analysis, the nonreducing sugars were subjected to acid hydrolysis with hydrochloride acid. These sugars were converted into reducing sugars, which were further subjected to titration and reduced the Fehling solution, according to the same method used for determination of reducing sugars. The results were expressed as grams of sugars per 100 mL of sample.

**Nonreducing Sugars**

The nonreducing sugars were estimated by subtracting the reducing sugars from the total sugars and multiplying this value by the conversion factor of glucose in sucrose (0.95). The results were expressed as grams of sucrose per 100 mL of sample.

### 2.2. GC-MS Analysis

Clove essential oil was subjected to detailed GC-MS analysis using a Shimadzu 2010 gas chromatograph fitted with an AB wax column as described previously [17, 18]. Helium was used as the carrier gas. A 0.1 mL sample was injected with splitless mode. The chemical components from the essential oil, shown in Table 1, were identified by comparing the retention times of chromatographic peaks with those of authentic compounds using the WILEY8.LIB and NIST05s.LIB.

### 2.3. Beverage Exposure to Teeth

The *in vitro* decalcification potential of the apple juice was evaluated using extracted human permanent teeth. The *in vitro *model was selected because the intact teeth surface is exposed to the beverage and the model allows for observation of erosion within a reasonable time period. The effect of the apple beverages to the healthy teeth was determined as described previously by Ehlen [19], with some modifications. Briefly, beakers were filled with 50 mL of apple juice, and the prepared fresh teeth from the volunteers were suspended in the beverages for a total of 24 hours at room temperature with constant stirring by using a magnetic stir bar. For comparison assays, clove oil and its lead molecules eugenyl acetate and eugenol was also introduced in the juices. After incubation period, the juices were analyzed for calcium contents using Inductively Coupled Plasma with Optical Emission Spectroscopy (ICP-OES) method according to standard reference method [20]. The exposure of the juice to teeth is, however, different than the regular drinking behavior of this juice, but the time intervals and frequent use of this juice is sufficient to cause dental erosions or decalcification. To eliminate inter- and intraoperator bias, data was analyzed “blindly” by third-party technicians unaware of the source of procured samples.

### 2.4. Inhibitory Action of the Clove Oil and Its Lead Molecules on the Decalcification of Teeth by the Apple Juice

To find out the inhibitory effect of the clove essential oil and its two lead molecules on the decalcification of the teeth by the apple juices, we added 0.05% (least toxic concentrations) of the clove oil and its lead molecules to the apple juices. The test solution containing 1000 ppm of fluoride solution as NaF serves as a positive control to prevent the decalcification of tooth. The teeth were suspended in the test compound added beverage for 24 hours at room temperature with constant stirring by using a magnetic stir bar. Calcium contents using Inductively Coupled Plasma with Optical Emission Spectroscopy (ICP-OES) method according to standard reference method [20].

### 2.5. Hemolytic Assay

Hemolytic activity was done as described previously [21]. The hemolytic activities of the clove essential oil, eugenol, and eugenyl acetate were determined on human red blood cells. Human erythrocytes from healthy individuals were collected in tubes containing EDTA as anticoagulant. The erythrocytes were harvested by centrifugation for 10 m at 2000 rpm at 20°C and washed three times in PBS. To the pellet, PBS was added to yield a 10% (v/v) erythrocytes/PBS suspension. The 10% suspension was then diluted 1 : 10 in PBS. From each suspension, 100 *μ*L was added in triplicate to 100 *μ*L of a different dilution series of the test compounds in the same buffer in eppendorf tubes. Fluoride was used as positive control. Total hemolysis was achieved with 1% Triton X-100. The tubes were incubated for 1 h at 37°C and then centrifuged for 10 m at 2000 rpm at 20°C. From the supernatant fluid, 150 *μ*L was transferred to a flat-bottomed microtiter plate (BIO-RAD, iMark, USA), and the absorbance was measured spectrophotometrically at 450 nm. The hemolysis percentage was calculated by following equation:

(3) %hemolysis=[(A450  of  test  compound  treated  sample  −  A450  of  buffer  treated  sample  A450  of  1%  TritonX-100  treated  sample  −  A450  of  buffer  treated  sample)]−  100%.  

## 3. Results

### 3.1. GC-MS Analysis

**Figure 1:**
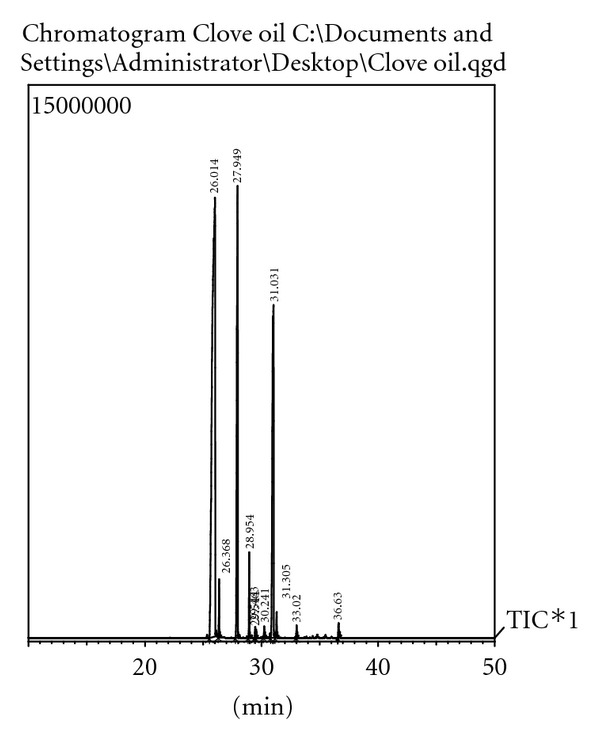
Chromatogram of clove essential oil showing representative peaks of eluted components. Peak at 26.014 min corresponds to presence of eugenol and peak at 31.031 min to eugenyl acetate. *X*-axis represents retention time (RT), and the *Y*-axis shows the intensity (abundance) of the signal.

GC-MS analysis of clove essential oil is shown in Table 1 and Figure 1. Eleven compounds representing 100% oil were identified, with the major components being eugenol (58.29%) and eugenyl acetate (19.10%).

Table 2 summarizes the results of the *in vitro* investigation of acidity (pH), titratable acidity (TA), reducing sugars (RS), nonreducing sugars (NRS), and total sugars (TS) mean values for the tested fruit juice. There were significant differences for the samples of apple juices for reducing, nonreducing and total sugars.

### 3.2. Decalcification Effect of Apple Juice

**Figure 2:**
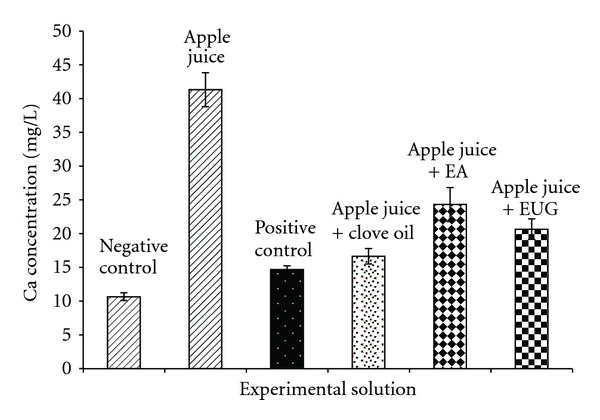
Calcium concentrations (mg/L) was measured for all groups, without apple juice as negative control (dark bar) and fluoride as positive control (white dots bar). Subsequently, test standard-apple juice only obtained after 24 hours incubation with the tooth (crossed bar), apple juice with clove oil incubated with tooth (black dots bar), apple juice with eugenyl acetate (EA) incubated with tooth (diamonds bar), and apple juice with eugenol (EUG) incubated with tooth (square bar). Measures were assessed using ICP-OES. Calcium was expressed as mg/L from the source and for test juice after swishing with and without teeth.

Analysis reveals consistent significant (*P* < 0.01 Student-*t*) increases in calcium leeched from teeth subjects after incubating with the tested apple juice. The ICP-OES measures were compared, and there were significant differences in calcium content of juice alone (11 ± 0.5) and juice incubated with tooth (41 ± 2.5), showing significant decalcification of teeth by the test juice (Figure 2). However, samples treated with clove essential oil and its two lead molecules showed significant decrease in the calcium content when compared to the control group. 1000 ppm fluoride was used as positive control for the inhibition of the decalcification of the apple juice and was observed to inhibit the decalcification to only 15 mg L^−1^ of calcium. Interestingly, clove essential oil observed to decrease the decalcification of tooth by the apple juice to only 17 mg L^−1^. Similarly, eugenol and eugenyl acetate also observed to inhibit the decalcification to 24 and 21 mg L^−1^, respectively. There was no statistically significant difference between the fluoride and clove oil treatment group. Out of the three test compounds, clove oil showed the major decalcification inhibition followed by eugenyl acetate and eugenol.

### 3.3. Hemolytic Activity

**Figure 3:**
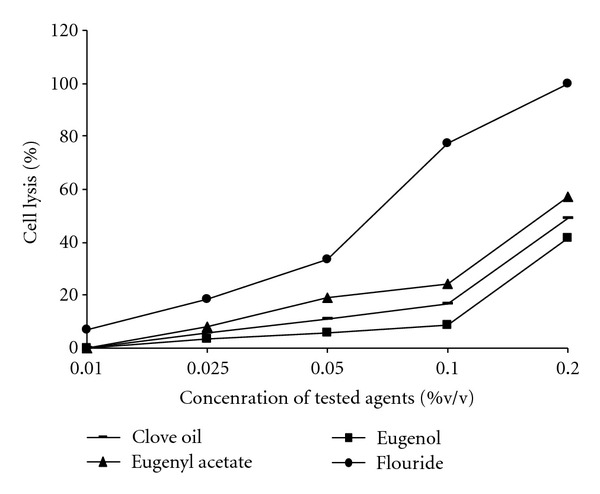
Hemolysis caused by different agents: clove oil, eugenol, eugenyl acetate, and fluoride. Hemolysis was determined by an absorbance reading at 450 nm and compared to hemolysis achieved with 1% Triton X-100 (reference for 100% hemolysis). The data are means of triplicate experiments.

Effects of clove oil, eugenol, eugenyl acetate, and fluoride on human red blood cells are shown in Figure 3. At 0.2% v/v, clove oil, eugenol, and eugenyl acetate showed 48, 41, and 57% hemolysis while fluoride at the same concentration showed 100% hemolysis. At the concentration of 0.05% of the test compounds, which had profound effect on the decalcification caused by the apple juice, only 11, 7, and 19% hemolysis was observed. This indicates that the clove essential oil as well as its lead molecules has low cytotoxic activity.

## 4. Discussion

The pH and buffering do not fully explain the erosive potential of the apple juices, as the mineral content, concentrations of organic acids (phosphoric and citric), and the ability of the mix to remove calcium from the mineral surface are all relevant. However, pH-expressing acid content, buffering ability, and acidic ions available for the overall general mordant effect of acid beverages are more important in producing erosion, as without an acid environment the other stated ions are not active. Table 2 shows how low the pH of the apple juice is, registering pH values well below the critical pH 5.5, at which tooth decalcification occurs. These results are noteworthy as the low pH values reflect that the apple juices are extremely acidic and consequently could all contribute to the decalcification of teeth (Figure 2). This indicates that not only are the apple juices highly acidic when they are first exposed to teeth, but they would also require a large amount of alkaline-stimulated saliva to be neutralized. The chemical composition of the apple juice had high buffering capacities and acid activity (pH) well below the critical pH 5.5. But when accounting for any extra calcium released, the calcium found in all the tested samples is consistently and significantly higher when compared to the calcium content in the apple juice alone (negative control). Tooth erosion, as chemical dissolution of calcium, is reflected by an increased content of calcium in all the tested samples. However, the use of the 0.05% clove oil, eugenol, and eugeyl acetate stop calcium dissolution by the apple juices after swishing (as shown in Figure 2). 1000 ppm fluoride was used as positive control as fluoride was found effective in reducing the erosions produced by various agents [22]. It is well documented that fluoride's anticariogenic effects take place via two principal mechanisms: inhibiting dental erosion when fluoride is present at the crystal surfaces during an acid challenge and enhancing remineralization by forming a low solubility veneer similar to the acid-resistant mineral fluorapatite (FAP) on the remineralized crystals [23]. Fluoride in this study was employed as a positive control, which was found to be effective in the process of dental erosion caused by the apple juices. Fluoride treatment inhibited the decalcification of teeth (Figure 2). 

The clove essential oil used in the present study consisted mainly of eugenol and eugenyl acetate. These phenolic compounds are potent antioxidants known to possess antimicrobial, local antiseptic, anesthetic [24], and anti-inflammatory and immunostimulating effects [25]. When mixed with zinc oxide, eugenol forms a material which has restorative and prosthodontic applications in dentistry. However, the effect of these compounds on the dental erosion and demineralization of dental hard tissues are less well understood [26]. Based on data obtained from our study, clove oil and its two active principles may positively affect the dental erosion process of apple juice through distinct mechanisms. In this study, clove-oil-treated teeth showed decreased decalcification with respect to control. The similar effect was shown by fluoride, which is a well-known inhibitor of the decalcification. Thus the potential inhibition of the erosion of the teeth by the clove oil and its lead molecules may also be attributed in the same way as was done by the fluoride. However, the higher concentrations of fluoride would be undesirable, as it would be unacceptably toxic and also negatively affect organoleptic taste properties [27]. To exclude the possibility of associated cytotoxicity of the clove oil and its derivatives, haemolytic activity on human erythrocytes was studied. Human red blood cells provide a handy tool for toxicity studies of the compounds, because they are readily available, their membrane properties are well known, and their lysis is easily monitored by measuring the release of hemoglobin [28]. The *in vitro* hemolytic assay is a possible screening tool for gauging *in vivo *toxicity to host cells [29]. The comparative study of the clove oil and its lead principles, eugenol and eugenyl acetate with the conventionally used fluoride indicate that these test compounds were significantly less cytotoxic. 

Based on data obtained in this *in vitro* study, we believe that the clove essential oil and its two lead molecules inhibit the decalcification and/or may promote the remineralization caused by the apple acidic beverages. The inhibition of the decalcification of the test compounds appears to be distinct like that of fluoride treatment. Unanswered concerns exist about the *in vivo* efficacy of clove essential oil and its lead molecules. Future research towards this objective, based on animal models, may resolve these issues.

## Figures and Tables

**Table 1:** GC-MS analysis of essential oil of clove.

Peak	Compound	Retention time	% of total
1	Eugenol	26.014	58.29
2	Alpha-Copaene	26.368	1.14
3	Beta-Caryophyllene	27.949	17.81
4	Alpha-Humulene	28.954	1.77
5	Delta-Cadinene	29.463	0.21
6	Alpha-Amorphene	29.544	0.16
7	Alpha-Farnesene	30.241	0.27
8	Eugenyl acetate	31.031	19.10
9	Alpha-Cubebene	31.305	0.53
10	Caryophylene oxide	33.020	0.26
11	Alpha-(E)-Ionone	36.630	0.45

**Table 2:** Mean values of apple juice for endogenous pH, titratable acidity (TA), reducing sugars (RS), nonreducing sugars (NRS), and total sugars (TS).

Fruit juice	pH	TA (% citric acid)	RS (g/100 mL)	NRS (g/100 mL)	TS (g/100 mL)
Apple	3.2	0.43 ± 0.02	4.84 ± 0.05	3.52 ± 0.04	8.36 ± 0.03
